# Microbial Diversity of Soil in a Mediterranean Biodiversity Hotspot: Parque Nacional La Campana, Chile

**DOI:** 10.3390/microorganisms12081569

**Published:** 2024-07-31

**Authors:** Carolina Quinteros-Urquieta, Jean-Pierre Francois, Polette Aguilar-Muñoz, Roberto Orellana, Rodrigo Villaseñor, Andres Moreira-Muñoz, Verónica Molina

**Affiliations:** 1 Programa de Doctorado Interdisciplinario en Ciencias Ambientales, Universidad de Playa Ancha, Valparaíso 2340000, Chile; carolina.quinteros@alumnos.upla.cl; 2 Departamento de Ciencias y Geografía, Universidad de Playa Ancha, Valparaíso 2340000, Chile; jpfrancois@upla.cl (J.-P.F.); polette.aguilar@upla.cl (P.A.-M.); roberto.orellana@upla.cl (R.O.); 3 HUB AMBIENTAL UPLA, Universidad de Playa Ancha, Valparaíso 2340000, Chile; villasenor.castro@gmail.com; 4 Centro de Investigación Oceanográfica COPAS COASTAL, Universidad de Concepción, Concepción 4070386, Chile; 5 Instituto de Geografía, Pontificia Universidad Católica de Valparaíso, Valparaíso 2362807, Chile; andres.moreira@pucv.cl

**Keywords:** bacteria, archaea, fungi, sclerophyllous, xerophytic, semi-arid, topoclimatic

## Abstract

Parque Nacional La Campana (PNLC) is recognized worldwide for its flora and fauna, rather than for its microbial richness. Our goal was to characterize the structure and composition of microbial communities (bacteria, archaea and fungi) and their relationship with the plant communities typical of PNLC, such as sclerophyllous forest, xerophytic shrubland, hygrophilous forest and dry sclerophyllous forest, distributed along topoclimatic variables, namely, exposure, elevation and slope. The plant ecosystems, the physical and chemical properties of organic matter and the soil microbial composition were characterized by massive sequencing (iTag-16S rRNA, V4 and ITS1-5F) from the DNA extracted from the soil surface (5 cm, *n* = 16). A contribution of environmental variables, particularly related to each location, is observed. Proteobacteria (35.43%), Actinobacteria (32.86%), Acidobacteria (10.07%), Ascomycota (76.11%) and Basidiomycota (15.62%) were the dominant phyla. The beta diversity (~80% in its axes) indicates that bacteria and archaea are linked to their plant categories, where the xerophytic shrub stands out with the most particular microbial community. More specifically, Crenarchaeote, Humicola and Mortierella were dominant in the sclerophyllous forest; Chloroflexi, Cyanobacteria and Alternaria in the xerophytic shrubland; Solicoccozyma in the dry sclerophyllous forest; and Cladophialophora in the hygrophilous forest. In conclusion, the structure and composition of the microbial consortia is characteristic of PNLC’s vegetation, related to its topoclimatic variables, which suggests a strong association within the soil microbiome.

## 1. Introduction

There is a close relationship between vegetation landscape and soil microbiome structures [1]. The co-occurrence of changes both in the plant community and soil microbiome composition and structure, along environmental gradients, suggest common associations or mechanisms between landscape function and morphology [2]. The soil is composed of extraordinarily diverse microbial communities [3], including different domains of life, both eukaryote (i.e., algae, fungi, protozoa) as well as prokaryote (i.e., bacteria and archaea) [4], the structure of which is associated with changes in environmental properties [5]. Among these properties, abiotic factors, such as pH, nitrogen availability [6], soil organic carbon content [7], temperature [8], redox condition [9] and also the physical soil structure, stand out [10]. Conversely, interactions, competition and predation by viruses and other grazers, besides plant communities, are biotic factors that influence the soil microbiome [5]. Additionally, there are hotspots characterized by an increased (heterotrophic or autotrophic) metabolic activity associated with the availability of built-up particulate organic matter [11], such as animal manure [12] or the rhizosphere [13]. The integration of this information can help us better understand the ecosystem’s functioning, not only regarding the soil as a natural habitat of microbial communities, but also regarding the way in which it influences the composition of the flora and fauna inhabiting such areas.

In Chile, studies on soil microorganisms provide relevant information on aspects such as vegetation productivity and fungal activity, mainly in the Crop–Livestock–Forestry area [14,15,16]. Other studies address prokaryote (i.e., bacteria and archaea) adaption and evolution processes in extreme environments [17,18], whether living freely [19] or associated with other organisms [20]. Research on ecosystems with different environments and climates, fluctuating between arid and temperate, represent significant changes in the soil microbiome related to environmental variables varying across the spatial scale [21,22]. Finally, changes in the microbial communities’ structure involved in pedogenic processes indicate clear upward (Proteobacteria, Acidobacteria, Chloroflexi, Verrucomicrobia and Planctomycetes) and downward (Actinobacteria and Gemmatimonadetes) trends of complete phyla along the north–south bioclimatic gradient [23]. These variations are partly the result of the relationship between climate and environmental factors (e.g., pH, plant-available P, organic matter, apparent density and clay %) and are strongly related to vegetation structure and composition. Thus, while an increased abundance of the so-called pioneer bacteria associated with pedogenic processes was observed in arid climates, an increased abundance of bacteria specialized in recycling organic compounds as a source of plant nutrients and soil stabilization was observed in arid and humid climates, which accounts for the impact of climate and slope on the microbial communities’ composition and structure [23].

The biodiversity hotspot, a term mainly used in conservation studies, is addressed from different biological criteria, such as endemism, which highlights its delimited distribution zones [24], species richness and the presence of rare species and their corresponding threat level [25], following the principles of vulnerability and irreplaceability in conservation planning. In this context, the definition based on the endemism criteria [24] for the biodiversity hotspot in Central Chile [26,27,28] is used. This region spans 40% of continental Chile, and the percentage of endemic vascular plants is similar to that of the California Floristic Province hotspot [29]. The aim of this study was to examine the composition and structure of microbial communities of diverse dominions of life (bacteria, archaea and fungi), in the soil of a representative area known as the biodiversity hotspot of Central Chile: Parque Nacional la Campana [26,27,30].

In particular, from the toposequence approach, we anticipate that the soil microbial community structure will be associated with sunlight exposure and elevation gradients with a higher diversity in the dry sclerophyllous forest and hygrophilous forest compared with xerophytic shrubland, with distinctive rare bacteria and archaea at the phyla level (<0.1%) and specific fungi. Consequently, high biodiversity at the bulk soil scale and its potential implications for the processes and functions associated with the vegetation landscape would be evident.

## 2. Materials and Methods

### 2.1. Study Area

**Figure 1:**
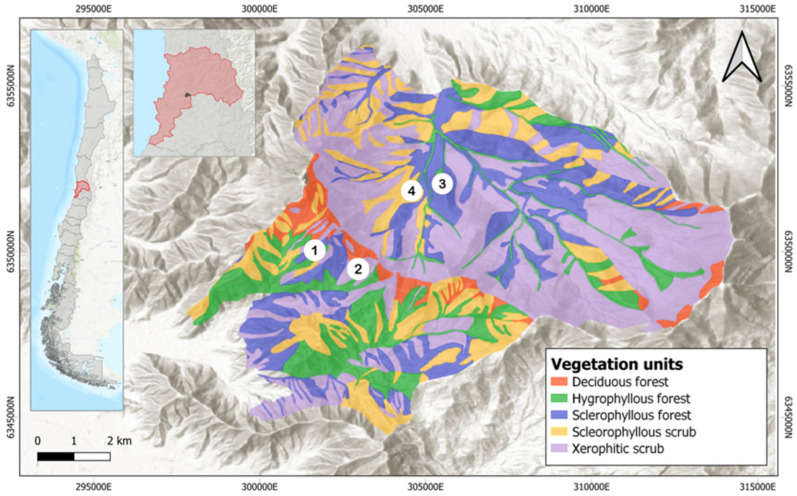
Map that shows PNLC sampling locations (1, 2, 3, 4). Vegetation map, adapted from Hauck et al. [38].

Parque Nacional La Campana (La Campana National Park) (PNLC), located in the Valparaíso region (32°55′–33°01′ S; 71°09′–71°01′ O) (Figure 1), was established as National Park in 1967 and as a National Biosphere Reserve in 1987 [31] as it stands out due to its good ecosystem conservation. The park, in the Coastal Cordillera of Central Chile, covers an area over 8000 ha and includes foothills, such as Cerro La Campana (1828 masl), which favors the development of topoclimatic conditions (e.g., elevation, slope, orientation, sunlight exposure, etc.) that enable the coexistence of ecosystems characteristic of the biodiversity hotspot of Central Chile [32]. Among them, the development of xerophytic and sclerophyllous shrubland and relict palm and oak forests with significant biogeographic relevance stands out [30,33].

PNLC has a Mediterranean climate with mean annual temperatures of 18 °C, and a rainy (480 mm per year) and mild winter which contrasts with a hot and dry summer [34]. Vegetation responds to the climate by developing sclerophyllous plant communities [35] and with a significant number of native and endemic species [36], subject to the particular topoclimatic and edaphic conditions [37]. Hence, hygrophilous communities are found in the valley floor forming peumo (*Cryptocaria alba*) and boldo (*Peumus boldus*) forests or swamp forests of myrtaceas (*Luma chequen*) and canelo (*Drymis winteri*). Sclerophyllous communities, which vary depending on the slope exposure and insolation, develop in average elevations (400–1100 masl). Thus, while quillay (*Quillaja saponaria*) and litre (*Lithraea caustica*) forests grow under the conditions of the sclerophyllous forests found in the south-facing slopes, plant communities characteristic of xerophytic shrubland, such as matorral de trevo (*Retanilla trinervia*) and succulents, are found on the north-facing slopes. In addition, there is a strong presence of Chilean palm trees (Juabea chilensis), which are part of various plant communities [38,39] in the northern sector of the PNLC [25,34]. Finally, subalpine communities characterized by oak forests (*Nothofagus macrocarpa*) and neneo (*Mulinum spinosum*) shrublands are found in sectors at higher elevations (1100–2000 masl) [37,38,39].

These plant communities present in PNLC also grow in soils with different physicochemical characteristics. For instance, the soil in the areas where xerophytic formation prevails has a pH and TOC (total organic carbon content) (6.0 and 3.1%) values different to those where the sclerophyllous forest (5.7 and 18.7%) formations develop [22]. Additionally, edaphic microbial communities, which have a fundamental role in contributing to nutrient recycling and plant growth through a symbiotic relationship with mycorrhizal fungi, are within the least studied soil components in Cordillera de la Costa [5].

### 2.2. Study Sites and Sample Collection

**Figure 2:**
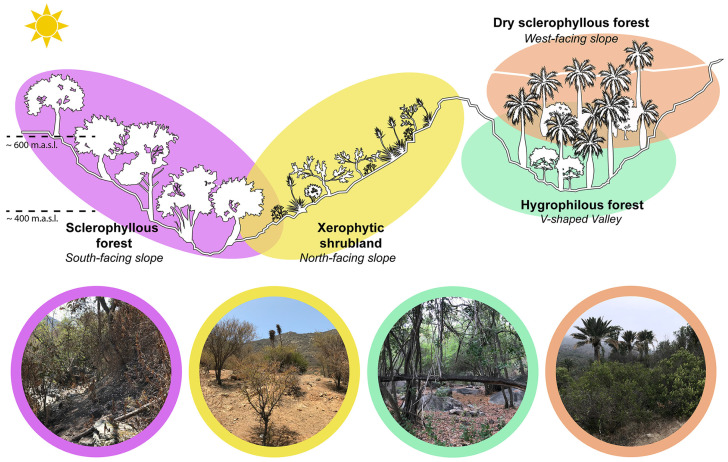
Graphical representation of the four sample sites characterized by their exposure and elevation.

The study sites (Figure 1) were selected on the basis of the relief and vegetation characteristics, and four sample sites, corresponding to plant communities, were established: (i) sclerophyllous forest on the southern slope, (ii) xerophytic shrubland on the north-facing slope, (iii) hygrophilous forest of *Drimys winteris* [38] and the presence of *Jubaea chilensis* at the bottom of the ravine and (iv) dry sclerophyllous forest on the west slope (Figure 2).

The sites were visited for two consecutive years in the austral summer (January 2020, Cajón Grande sector, and February 2021, Palmas de Ocoa sector), and the geographic coordinates, altitude, slope, orientation and type of dominant plant community were registered for both of the sites (Figure 2, Table 1). In the latter case, an analysis of the plant species composition and cover of each sampling site was conducted by way of the standard phytosociological methodology [40]. To do this, a plant census of each study site was carried out in 10 × 10 m^2^ plots, and a list of the species present and an estimate of the relative abundance of the number of individuals coverage per plot was recorded. For cover values of less than 1%, the signs “+” and “r” (cross and r) were used; the former when various individuals of the species in question were found, and the latter when there was just one [41,42]. The results were expressed as a percentage of the herb, shrub and tree layer cover. A census of species such as peumo (*Cryptocarya alba*), boldo (*Peumus boldus*), quillay (*Quillaja saponaria*) and palito negro (*Adiantum chilense*), among others, with closed forest aspect and the presence of litre (*Lithraea caustica*) in the surroundings, was conducted in the sclerophyllous forest. Species such as litre (*Lithraea caustica*), trevo (*Retanilla trinervia*), espino (*Acacia caven*) and puya (*Puya chilensis*), of the thorny type were observed in the xerophytic shrubland. Boldo (*Peumus boldus*), quillay (*Quillaja saponaria*) and litre (*Lithraea caustica*) were found in nearby areas. A census of canelo (*Drimys winteri*) and Chilean palm tree (*Jubaea chilensis*) forest, among others, with closed forest aspect, was conducted in the hygrophilous forest. A census of species of Chilean palm trees (*Jubaea chilensis*), litre (*Lithraea caustica*), colliguay (*Colliguaja odorífera*) and bollén (*Kageneckia oblonga*) was conducted in the dry sclerophyllous forest. The latter four species are characteristic of shrub-like dry sclerophyllous forest [37]. Palito negro (*Adiantum chilense*) growing along the censused hillside was observed.

Soil surface samples (5 cm) were collected at random within 10 × 10 m^2^ plots [2]. Sterile Falcon tubes and gloves were used to prevent the contamination of the samples. A total of 4 representative samples (i.e., plots) were taken from each site (Table 1). After the samples were collected, they were transported to the laboratory in cold gel packs, then they were homogenized and subdivided for analysis; part of the samples was stored at 4 °C for physicochemical analysis (pH, LOI) and the remaining part was stored in cryotubes, in an RNAlater solution [2], at −20 °C for isotopic and molecular analysis.

### 2.3. Climate Variables in PNLC

Climate variables (e.g., mean annual temperature, annual precipitation, etc.), associated with the sampling sites were obtained from 2 complementary databases. The first one, the WorldCLim 2.1 database [43], contains average values gridded data for the period of 1970–2000 from 1 km^2^; the second one corresponds to the CR2 gridded data [44], with a spatial resolution of 5 km^2^, for the period of 1979–2016. Thus, and using the SIG ArcGis platform, version 9.3, and Global Mapper for each sampling point, an average value of the applied climate variables was obtained (Figure 1, Table 1). Supplementary Table S1 shows all the measured data.

### 2.4. Soil and Organic Matter Physicochemical Analysis

In order to determine soil parameters, such as pH, dry density, organic content, C/N ratio, and δ15N δ13C isotope values, subsamples representative of each site were analyzed. The dry density and moisture content were determined using the LOI method, drying the samples at 105 °C for 24 h [45]. The soil pH was determined following the INIA-recommended process [46], consisting of resuspending 20 g of dry soil in 50 mL of water, using a magnetic stirrer at a controlled temperature of 23 °C for 5 min and then allowing it to settle for 3 h. The pH was measured with a Thermo Scientific Orion Star A215 X11399 multiparametric meter (Thermo Fisher Scientific, Waltham, MA, USA) and pH electrode Thermo Scientific, Series VZ1-15288 (Thermo Fisher Scientific). The soil organic matter composition was determined by analyzing samples of 50 mg through an Isotope Ratio Mass Spectrometer, IRMS, at the Laboratory of Biogeochemistry and Applied Stable Isotopes of the Pontificia Universidad de Chile (LABASI). The results are shown in relation to the δ15N vs. air and δ13C vs. VPDB value.

### 2.5. DNA Extraction and Sequencing of Prokaryote and Fungal Phylogenetic Markers

The microbial composition was determined by first extracting the total genomic DNA from 250 mg of the soil using the MoBio PowerSoil^®^ DNA isolation kit (MO BIO Laboratories, Inc., Carlsbad, CA, USA), following the manufacture’s protocol. Subsequently, DNA quantification was performed using the Qubit fluorometer (Thermo Fisher Scientific), and its amplification was verified by conventional PCR. The variable V4 region of the 16S rRNA gene was sequenced through high-throughput sequencing using primers 515F (GTGCCAGCMGCCGCGGTAA) and 806R (GGACTACHVGGGTWTCTAAT) [47] for bacteria and archaea and with region ITS1-5F, with primers ITS5-1737F (GGAAGTAAAAGTCGTAACAAGG) and ITS2-2043R (GCTGCGTTCTTCATCGATGC) for fungi [48]. This was performed using the NovaSeq PE250 sequencing platform (Novogene Biology Information Technology Co., Ltd., Beijing, China). The sequences were entered into the ENA PRJEB56868 database.

### 2.6. Analysis of Microbial Composition and Other Statistic Data

Paired-end sequences were imported and aligned from the “manifiest Fastq” format using QIIME2 2021.4 [47]. The sequences were subjected to quality filtering and chimera checking, leaving prokaryotes with a length between 206 and 312 bp and fungi with a length between 218 and 424 bp, using the QIIME2 DADA2 denoise-paired plugin [49] (see Supplementary Table S2). Then, microorganism sequences in amplicon sequence variants (ASV) [50] were classified using the Greengenes database version 13_8 at 99% [51] of the 515F/806R sequence region and the UNITE QIIME database release for fungi for ITS regions with a threshold at 99% [52,53].

The microbial community was analyzed in RStudio v4.2.2 software using a ‘phyloseq’ package [54]. First, the libraries were rarefied using the rarefy_even_depth function, and the alpha diversity indices (richness, Shannon index, Pielou’s evenness index) were determined with the estimate_richness and evenness functions. Venn diagrams were drawn using the get_vennlist function of the ‘MicrobiotaProcess’ package at the ASVs level [55] to identify the microbial community composition. Changes in the microbial community at the phylum level were analyzed using the amp_heatmap and amp_boxplot functions of the ‘ampvis2’ package [56].

In order to identify differences in the microbial community structures among the study sites, the Bray–Curtis distance was estimated, and its variation was analyzed by Principal Coordinate Analysis (PCoA) using the ordinate function of the ‘phyloseq’ package. Likewise, the level of association between environmental factors and changes in the microbial community (at the phylum level) was determined using the envfit function in the ‘vegan’ package and was graphed on the PCoA. A differential analysis using the ASV was performed with the ‘DESeq2’ package [57], from non-normalized libraries, to determine the microorganisms that varied significantly between the sites. The potential functions associated with the ASVs present in the different samples were determined by using the trans_func function of the ‘microeco’ package [58], by specifying the FAPROTAX database [59] for prokaryotes and FUNGuild [60] for fungi. The results were plotted using the plot_spe_func_perc function of the same package.

A redundancy analysis (RDA) was carried out on environmental variables, and accomplished with the rda function of the ‘vegan’ package [61]. The differences between environmental parameters and alpha diversity in the sites were analyzed using a One-Way Analysis of Variance (ANOVA) followed by a post hoc (Tukey’s HSD) test, ensuring that the ANOVA assumptions (Shapiro test of normality and Levene’s test of homoscedasticity) were complied with; otherwise, a non-parametric Kruskal–Wallis test was performed, using the functions aov and TukeyHSD included in the ‘stats’ package.

## 3. Results

### 3.1. Environmental and Soil Physicochemical Conditions

**Figure 3:**
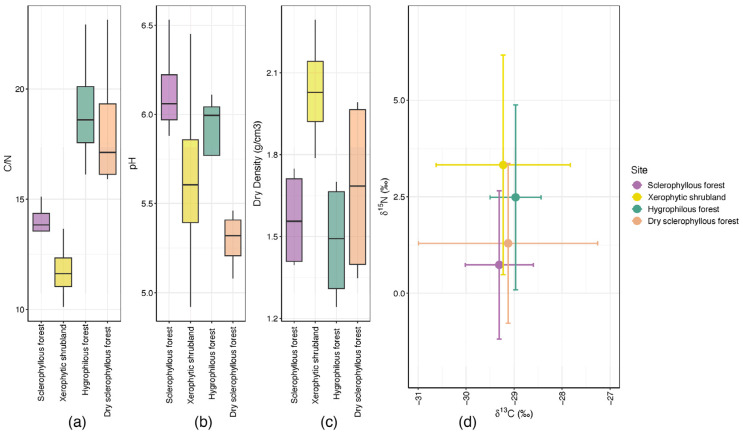
Physicochemical variables of the soil samples: (**a**) C/N. Significant C/N differences were observed between the hygrophilous forest and the xerophytic shrubland, and between the dry sclerophyllous forest and the xerophytic shrubland. (**b**) pH. No significant differences between locations were observed. (**c**) Dry density (g/cm^3^). Significant differences were observed between the hygrophilous forest and the xerophytic shrubland (**d**) d15N and d13C isotope content.

The environmental conditions in the study sites in Parque Nacional La Campana very well represent the so-called Mediterranean climate with an oceanic influence. There are slight variations in the climate parameters, precipitation and temperature within the locations. Exposure is, perhaps, the most significant factor (as shown in Supplementary Table S1). Regarding the plant cover, significant tree cover at the hygrophilous forest (95%), sclerophyllous forest (85%), dry sclerophyllous forest (70%) and xerophytic shrubland (60%) were observed. Regarding the annual temperature range, an increased variation in temperature between the warmest and coldest day in the hygrophilous forest and dry sclerophyllous forest, which are at a highest latitude, was observed. Annual precipitation ranged from 353 to 371 mm (Supplementary Table S1). The soil physicochemical analysis reported that the average pH was lower in the dry sclerophyllous forest (5.295), while it was higher (Figure 3b) in the sclerophyllous forest (6.1325 g/m^3^). Regarding the dry density, the lowest average was recorded in the dry sclerophyllous forest (42.7 g/m^3^) and the highest in the xerophytic shrubland (159.2 g/m^3^) (Figure 3c). A carbon and nitrogen isotope composition showed the highest values in the xerophytic shrubland in comparison with the sclerophyllous forest, which shows the lowest values (Figure 3d). The sites in the hygrophilous forest and dry sclerophyllous forest had similar N values and a higher C content.

**Figure 4:**
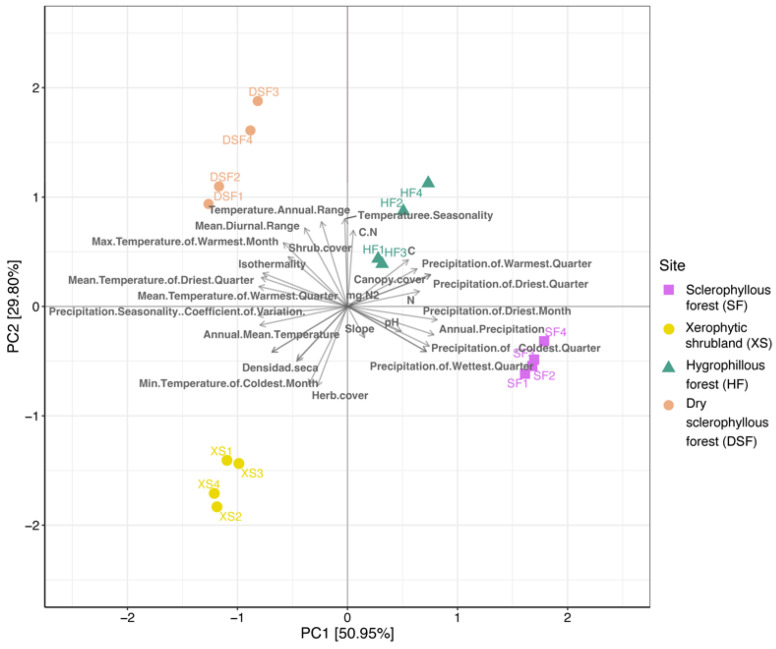
Redundancy analysis (RDA) that shows the variability of the environmental conditions associated with the plant communities. (For further information, see Supplementary Table S3.)

The PCA shows the environmental variability of the study sites associated with the climate conditions, the soil physicochemical characteristics and plant cover, which evidences a clear difference associated with the plant communities (Figure 4). The slopes were associated with different temperature and precipitation components, plant cover and soil conditions that, in total, represent >80% of the variability. For instance, the xerophytic shrubland site is associated with the mean annual temperature variation, herb cover and dry density, while the sclerophyllous forest is mainly associated with the annual precipitation, and precipitation in the driest, coldest quarter and more humid months. In the sites of the dry sclerophyllous forest and hygrophilous forest, it is associated with temperature, precipitation and soil carbon content.

### 3.2. Sequencing and Structure of the Soil Microbial Community

The number of sequences obtained from the soil microorganisms of PNLC, after the sequencing and the curing process, is summarized in Table S2. A larger number of fungal sequences was observed, with an average of 141.898 (fungi), compared to 93.706 (prokaryotes), reaching an adequate recovery of the expected microbial richness based on the rarefaction curves (Supplementary Figure S1 for fungi and Figure S2 for prokaryotes).

**Figure 5:**
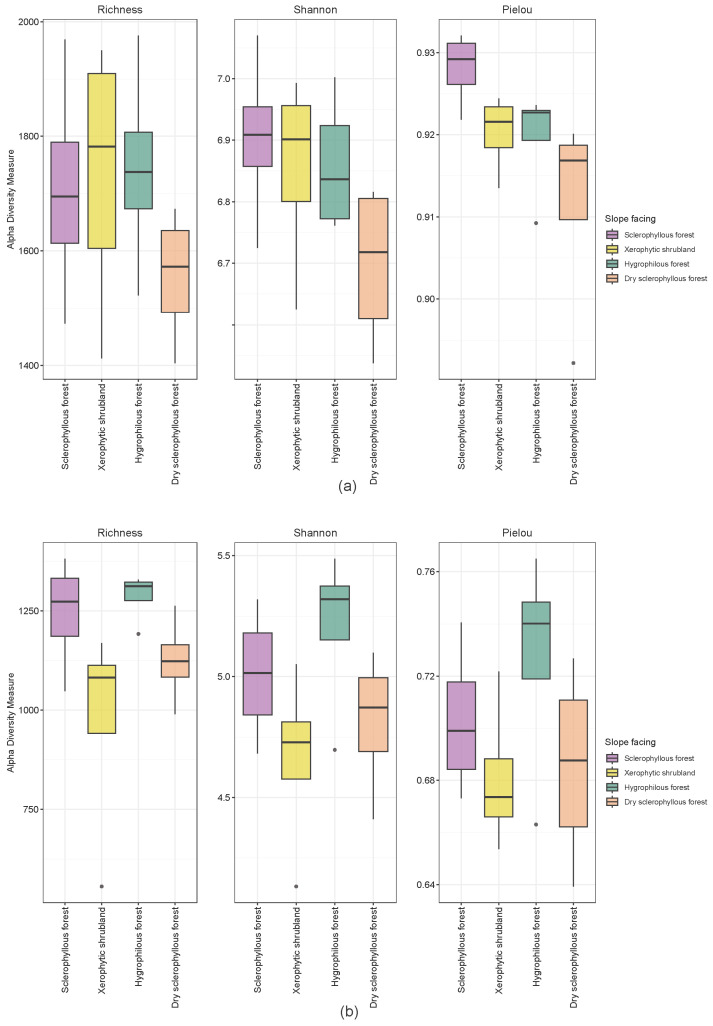
(**a**) Prokaryote and (**b**) fungi soil microorganism’s alpha diversity in PNLC plant communities. Grey dots correspond to replicate outliers.

The estimated soil prokaryotic and fungal richness and diversity are shown in Figure 5a,b, respectively. In the case of prokaryotes, a higher richness and composition diversity in the sclerophyllous forest, xerophytic shrubland and hygrophilous forest soils was observed, where the sclerophyllous forest presented a higher evenness, reaching significant differences in comparison with the dry sclerophyllous forest (Kruskal–Wallis test, *p*-value < 0.05). The hygrophilous forest soils presented a higher diversity and evenness in fungi composition in comparison with other sites.

**Figure 6:**
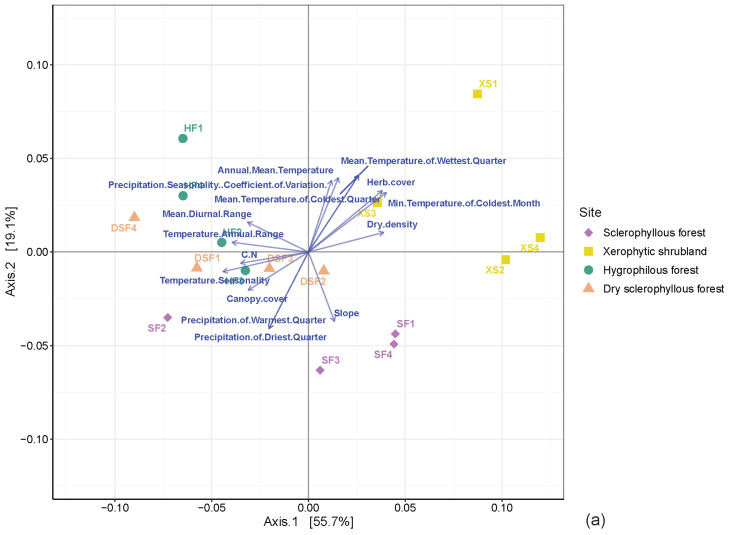

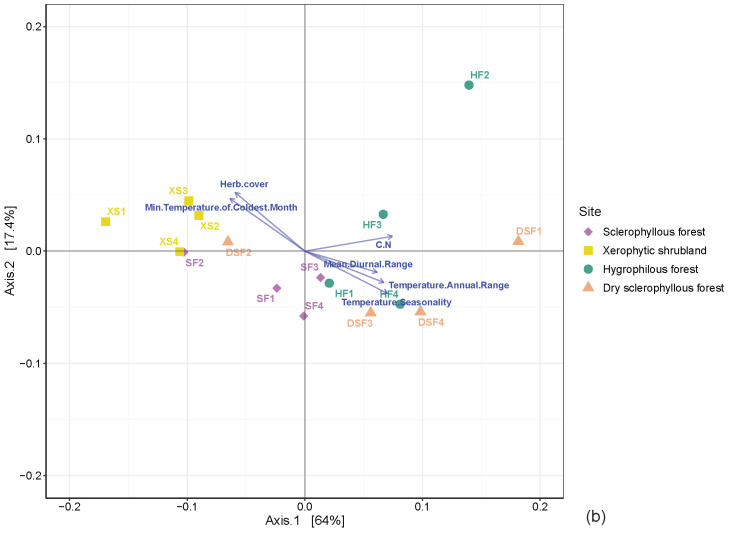
PCoA (PERMANOVA *p*-value < 0.05) that illustrates the microbial variability of the soil of the plant communities at the phylum level of (**a**) prokaryotes and (**b**) fungi. The blue arrows represent the environmental parameters that significantly correlate with the microbial communities (envfit, *p*-value < 0.05).

The variability of the microbial structure based on the beta-diversity analysis at the phylum level for prokaryotes and fungal communities is visualized using PCoA, which represents 74.8% and 81.4% of the variance, respectively (Figure 6a,b). This result reveals that there is an intra-site clusters generation both for bacteria and archaea, as well as for fungi, given that environmental variables have a similar impact in both cases (where axis 1 has a 60% range and axis 2 is close to 20%). Firstly, it was established that there are significant differences between the communities of the different plant communities for prokaryotes and fungi (PERMANOVA, *p*-value < 0.05). Additionally, similarities have been observed between prokaryote communities of the hygrophilous forest and dry sclerophyllous forest soils. In accordance with the PCoA of fungal communities, the xerophytic shrubland community shows a microbial community with more similarities within it in comparison with the other analyzed soils. These variabilities were associated with climatic variables and soil physicochemical characteristics. For xeric environments (*p*-value < 0.05), herb cover and the minimum temperature of the coldest month were significant for prokaryotes and fungi. In the case of the bacteria–archaea communities in a sclerophyllous environment, the slope, precipitation of warmest quarter and precipitation of driest quarter were significant. Regarding the hygrophilous forest and dry sclerophyllous forest soils, the temperature annual range and temperature seasonality were significant for bacteria and archaea, as well as for fungi.

### 3.3. Microbial Composition in Different Types of Environments

**Figure 7:**
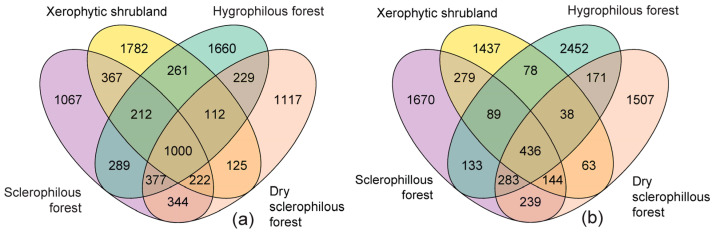
Venn diagram that shows the comparison of ASVs richness associated with (**a**) prokaryotes and (**b**) fungi, grouped on the basis of the soil of the plant communities. Sclerophilous forest—purple area, Xerophytic shrubland—yellow area, Hygrophilous forest—green area, Dry sclerophilous forest—orange area.

The Venn diagram below, Figure 7, allows the comparison of the soil microbiome of plant communities in PNLC between prokaryotes and fungi. Soil prokaryote communities show 1000 common (core) ASVs in all of the sites, representing 10.9% (Figure 7a). The xerophytic shrubland soil with 1782 ASVs showed a higher number of unique prokaryotes, followed by the hygrophilous forest with 1660 ASVs, dry sclerophyllous forest with 1117 ASVs and sclerophyllous forest with 1067 ASVs (Figure 7a). Fungi showed a bigger number of shared ASVs in all of the sites, 436 ASVs, which represent 4.8% of the total number of sequences (Figure 7b) compared with each plant community, where the hygrophilous forest concentrated the largest number of unique ASVs with 2452 sequences (27.1%), followed by the sclerophyllous forest, with 1670 ASVs, the dry sclerophyllous forest with 1507 ASVs and the xerophytic shrubland with 1437 ASVs (Figure 7b).

**Figure 8:**
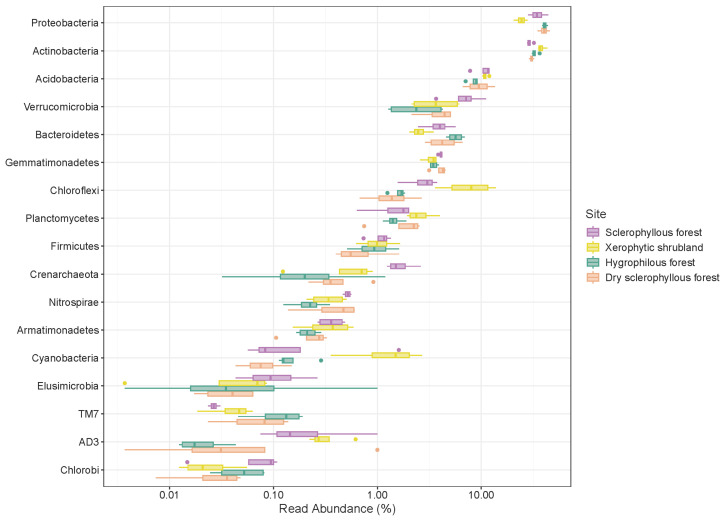
The box plot shows the relative abundance of bacterial and archaeal phyla in the PNLC soil categorized by the different plant communities.

For bacteria, 98.27–99.59% of the sequences (Figure 8) showed a dominance of prokaryotic microbial communities’ composition, particularly in the south-facing slopes (sclerophyllous forest) with a greater number of archaea (1.7%). In general, the most abundant bacterial phyla were the Proteobacteria (35.43%), Actinobacteria (32.86%), Acidobacteria (10.07%), Verrucomicrobia (4.55%), Bacteroidetes (4.24%), Gemmatimonadetes (3.75%) and Chloroflexi (3.66%), followed by Planctomycetes (1.92%), Firmicutes (0.99%) and the Crenarchaeote (0.80%) phylum of archaea. Among the low-abundance microorganisms (<0.5%), there was the presence of the bacteria Nitrospirae (0.49%), Armatimonadetes (0.43%), Cyanobacteria (0.69%), Chlorobi (0.07%), Elusimicrobia (0.07%), AD3 (0.17%), WS3 (0.06%), TM7 (0.08%) and WPS-2 (0.05%). The distribution of these phyla varied from site to site, especially for the less frequent groups < 1% and the rare biosphere < 0.1% (Figure 8).

In the xerophytic shrubland, the bacterial phyla Proteobacteria, Actinobacteria, Acidobacteria and Chloroflexi represent >10% of the total sequences, followed by Gemmatimonadetes, Bacteroidetes, Verrucomicrobia, Planctomycetes, Firmicutes and Cyanobacteria (1–10%). Crenarcheaote, Nitrospirae, Armatimonadetes and AD3 represent <1%, and at the rare biosphere level < 0.1%, the Chlorobi, Elusimicrobia and TM7 phyla are found. Proteobacteria, Actinobacteria and Acidobacteria are the most abundant bacterial phyla (>10%) in the sclerophyllous forest, followed by Gemmatimonadetes, Bacteroidetes, Verrucomicrobia, Chloroflexi, Planctomycetes, Firmicutes and Crenarchaeota (1–10%), Nitrospirae, Armatimonadetes, Cyanobacteria, Elusimicrobia and AD3 (0.1–1%). In comparison with other plant communities, there was a lower number of phyla of the rare biosphere (<0.1%) in the sclerophyllous forest (Chlorobi and TM7).

The bacterial phyla Proteobacteria and Actinobacteria (>10%) were the most abundant in the hygrophilous forest, followed by Acidobacteria, Verrucomicrobia, Bacteroidetes, Gemmatimonadetes, Chloroflexi, Planctomycetes and Firmicutes (1–10%). Crenarchaeote, Nitrospirae, Armatimonadetes, Cyanobacteria and Elusimicrobia consist of 0.1–1% of the sequences. The larger number of phyla classified as part of the rare biosphere (<0.1%), including Chlorobi, AD3 and TM7, are found in the hygrophilous forest. Additionally, there are only two abundant phyla > 10% (Proteobacteria, Actinobacteria and Acidobacteria) in the dry sclerophyllous forest. The bacterial phyla Gemmatimonadetes, Bacteroidetes, Verrucomicrobia, Chloroflexi, Planctomycetes and Firmicutes represent 1–10% of the sequences. Crenarchaeote, Nitrospirae, Armatimonadetes, Cyanobacteria and TM7 (0.1–1%) are more rarely found. The bacterial phyla Chlorobi, Elusimicrobia and AD3 (<0.1%) are found in the rare biosphere.

**Figure 9:**
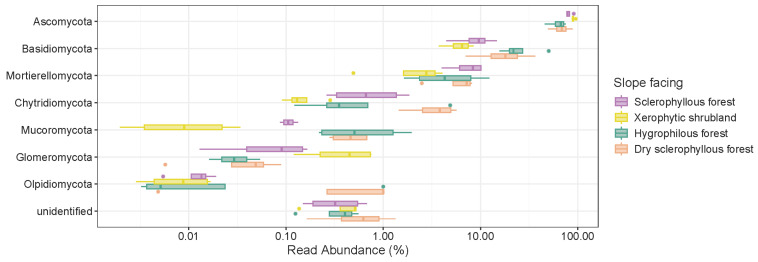
The box plots show the relative abundance of the fungal phyla in the PNLC soil categorized by different plant communities.

A smaller number of fungal phyla (Figure 9) were found in the PNLC soil. The phylum Ascomycota reached 76.11%, followed by Basidiomycota (15.62%), Mortierellomycota (5.63%) and Chytridiomycota (1.56%), and a new unidentified phylum was found (0.46%). There was a variability associated with less abundant and rare phyla among the different sampled sites. In the xerophytic shrubland, Ascomycota represent >10% of the microbial community while the phyla Basidiomycota and Mortierellomycota constitute <10%, and Chytridiomycota and Glomeromycota between 1% and >0.1%, respectively, of the microbial community. The phyla Mucoromycota and Olpidiomycota were found at the level of the rare biosphere (<0.1%). In the sclerophyllous forest, the most abundant fungal community (>1%) was similar to that in the xerophytic shrubland, while the less abundant fungal communities were associated with the phyla Glomeromycota and Olpidiomycota (0.1–1%). The phyla Ascomycota and Basidiomycota were more abundant (>10%) in the hygrophilous forest, followed by the phyla Mortierellomycota (1–10%), Chytridiomycota and Mucoromycota, (0.1–1%). As in the sclerophyllous forest, the rare biosphere is represented by Glomeromycota and Olpidiomycota (<0.1%). The same abundant phyla (>10%, Ascomycota and Basidiomycota) were observed in the dry sclerophyllous forest. The phyla Mortierellomycota and Chytridiomycota represent 1–10% abundance, followed by Mucoromycota and Olpidiomycota (0.1–1%), where Glomeromycota (<0.1%), as in the other sites, constituted the rare biosphere.

An unidentified phylum (0.1–1%) is present in all the sites. In addition, it was observed that fungal groups (e.g., Chytridiomycota and Mucoromycota) are more abundant than prokaryotes. This suggests that fungi might be site-specific communities.

### 3.4. Distribution of Microbial Communities among the Different Plant Communities

**Figure 10:**
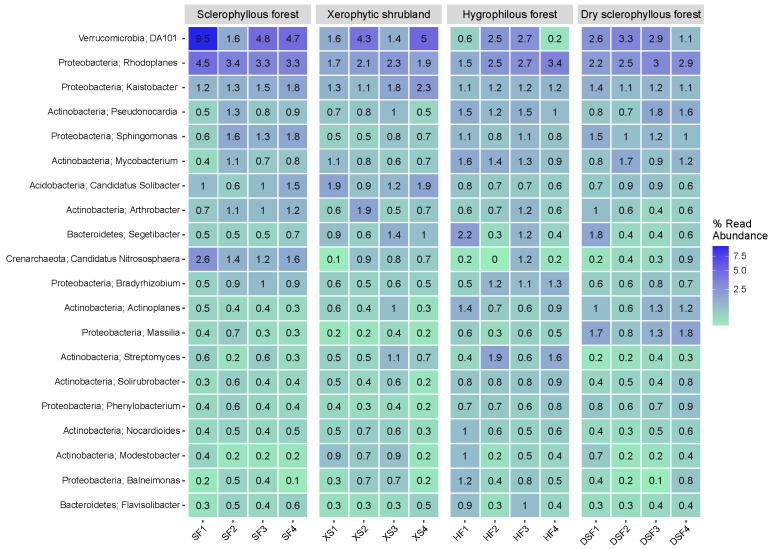
Heatmap of the 18 most abundant genera of prokaryotes in the soil (*n* = 16) analyzed in PNLC, categorized by plant communities.

The distribution of the 18 bacterial and archaeal genera that individually contribute a higher percentage to the soils is shown in Figure 10. *DA101* is the most abundant in the sclerophyllous forest plant community, whereas the genera *Rhodoplanes* and *Candidatus Nitrososphaera* also show a higher relative contribution. In addition, a number of genera represent individually >1% in the dry sclerophyllous forest in comparison with the sclerophyllous forest and xerophytic shrubland, such as *Pseudonocardia*, *Mycobacterium*, *Actinoplanes*, *Segetibacter* and *Massilia* (Figure 10).

**Figure 11:**
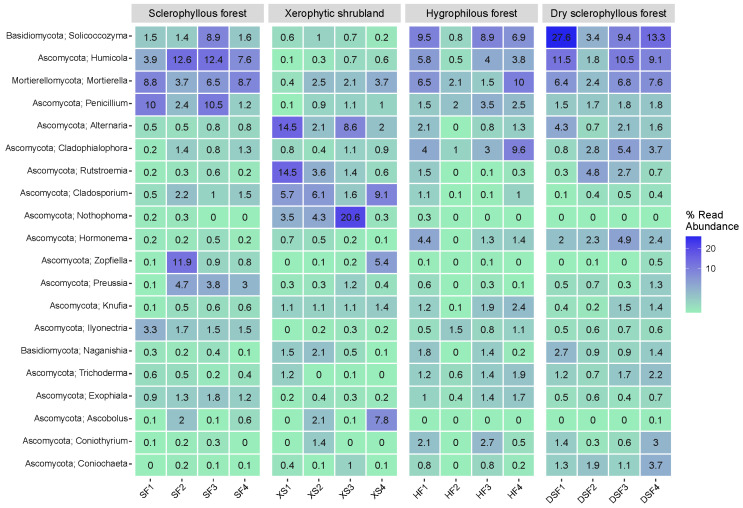
Heatmap for the 20 genera of fungi in the soil (*n* = 16) analyzed in PNLC categorized by plant communities.

Figure 11 shows the distribution of 20 genera of fungi that individually represent the highest percentage in the soil categorized by different plant communities. The genera *Solicoccozyma*, *Humicola*, *Mortierella*, *Penicillium* and *Zopfiella*, represent up to 10% in the sclerophyllous forest. The genera *Alternaria*, *Rutstroemia*, *Cladosporium*, *Nothophoma*, *Zopfiella* and *Ascobolus* presented higher values in the xerophytic shrubland. Sharing the presence of abundant genera associated with *Solicoccozyma* (26.8%) are *Humicola*, *Mortierella* and *Cladophialophora* in the hygrophilous forest and dry sclerophyllous forest. Some samples of the hygrophilous forest show a higher individual representation of the genera *Cladophialophora*, while genera such as *Hormonema* and *Coniochaeta* were far more abundant in the dry sclerophyllous forest (Figure 11).

### 3.5. Analysis of Differential Abundance of Microorganisms

**Figure 12:**
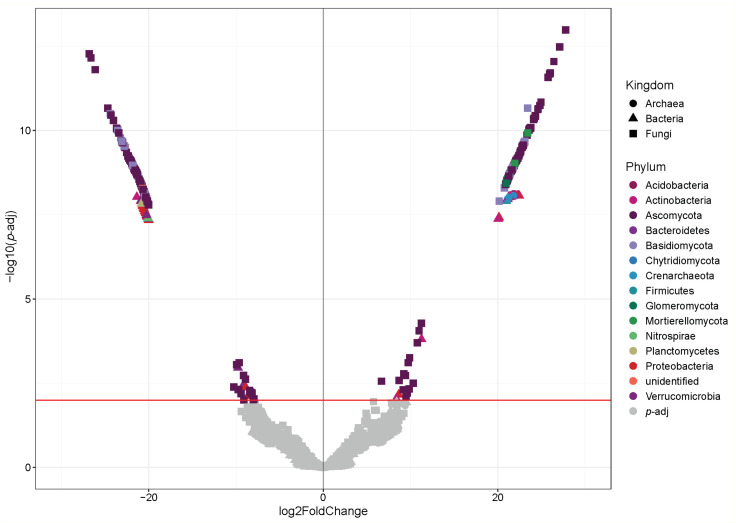
Volcano plot that shows the ASVs of microorganisms (bacteria, archaea and fungi) when comparing the xerophytic shrubland vs. sclerophyllous forest plant communities in PNLC. The red line indicates the significantly different ASVs (*p*-adj). Complete data in Supplementary Table S4.

The DeSeq analysis represented in a Volcano plot allowed the identification of differentially represented ASVs in the microbial communities in the soil of plant communities in the xerophytic shrubland versus sclerophyllous forest (Figure 12, Supplementary Table S4). Particularly, 335 ASVs among the sites are observed; 167 ASVs in the sclerophyllous forest and 168 in xerophytic shrubland. The community is composed of 16 phyla, where Ascomycota (199 ASV), Basidiomycota (47), Actinobacteria (23 ASV) and Proteobacteria (18) stand out.

Although these different taxa were found at both sites, they were distributed in a different manner; the phyla Ascomycota (107 ASV), Basidiomycota (32 ASV) and Proteobacteria (13 ASV) were more abundant in the sclerophyllous forest, while the phyla Actinobacteria (18 ASV) and Acidobacteria (3 ASV) were more abundant in the xerophytic shrubland.

**Figure 13:**
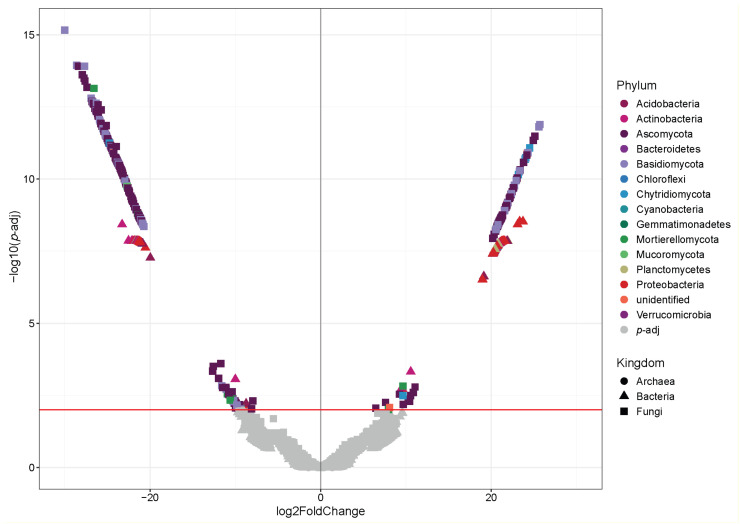
Volcano plot that shows the ASVs of microorganism when comparing the hygrophilous forest and dry sclerophyllous forest plant communities in PNLC. The red line indicates the significantly different ASVs (*p*-adj). Complete data in Supplementary Table S5.

When comparing the ASVs of the microbial communities in the hygrophilous forest and the dry sclerophyllous forest based on the DeSeq analysis represented in a Volcano plot (Figure 13), 563 ASVs were found to be significant *(p*-adj). The community is composed of 49 phyla, where Ascomycota (264 ASVs), Basidiomycota (100 ASVs), Proteobacteria (61 ASVs), Actinobacteria (47 ASVs), Acidobacteria (28 ASVs) and Gemmatimonadetes (11 ASVs) stand out. Regarding their distribution, the phyla Ascomycota (181 ASVs), Basidiomycota (72 ASVs), and Actinobacteria (25 ASVs) are more abundantly found in the dry sclerophyllous forest and Acidobacteria (15 ASVs), Gemmatimonadetes (9 ASVs) and Chytridiomycota (5 ASVs) in the hygrophilous forest.

### 3.6. Predicted Potential Microbial Function

**Figure 14:**
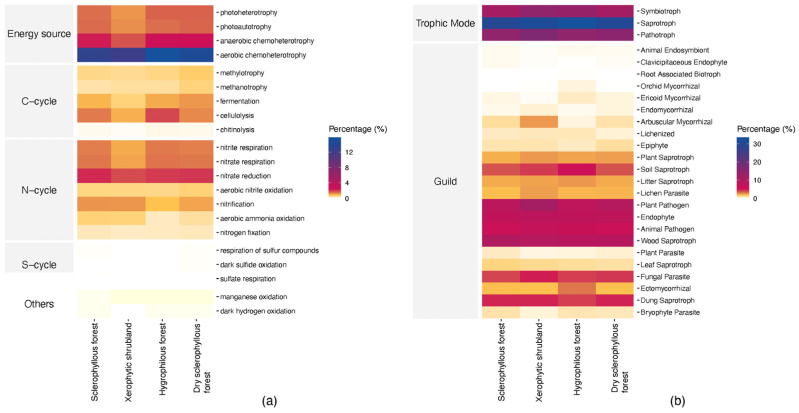
Predicted potential microbial function profile by comparison of the 4 study sites. (**a**) Prokaryotes, (**b**) fungi.

It was predicted that the potential functional profile microbial function of prokaryote communities (Figure 14a) would be similar in each site. Aerobic chemoheterotrophs, anaerobic chemoheterotrophs, photoheterotrophy and photo-autotrophy are predominant in obtaining energy; while nitrate reduction is associated with the nitrogen cycle, the cellulolytic bacteria are associated with the carbon cycle. Regarding fungi (Figure 14b), at a trophic level, saprotrophic fungi, followed by pathotroph and symbiotroph, are predominant in all sites; regarding ecological guilds, the plan pathogen, endophyte, animal pathogen and wood saprotroph stand out. Regarding each plant community, a slight increase in nitrifying prokaryotes is observed in the sclerophyllous forest, hygrophilous forest and dry sclerophyllous forest in comparison with the xerophytic shrubland; and, regarding fungi, an increase in the soil saprotroph in the sclerophyllous forest, fungal parasites in the xerophytic shrubland and dung saprotroph in the sclerophyllous forest, xerophytic shrubland and dry sclerophyllous forest is observed.

## 4. Discussion

### 4.1. Toposequence, Microbial and Plant Communities in PNLC

In a toposequence, the direction of the slope is a significant topographic factor that affects the local microclimate, influencing the solar radiation, soil temperature, water retention and availability and nutrient dynamics [62,63,64]. These conditions determine the plant distribution and potentially determine the soil microbial communities’ composition and activity, which has been reported for the north- and south-facing slopes in Chilean national parks, such as Pan de Azúcar, Santa Gracia, La Campana and Nahuelbuta, each of them with a different type of climate [21,22,23], and for the north and south slopes of the Italian Alps [2,65]. These studies, focused on climosequences and toposequences, have reported differences in the soil microbial communities’ [66,67] diversity, abundance and activity. In the Chilean national parks’ case, there was a comparative study conducted along the north–south climatic gradient of the coast of Chile which, among other things, accounts for changes in microbial abundancy and biodiversity (i.e., bacteria and archaea) in relation with the north–south arid–humid gradient [21,22]. Likewise, a study reveals the change in microalgae and cyanobacteria richness associated with the biocrusts along this gradient, where a lower species richness is observed in arid climates and a higher richness in Mediterranean climates [68].

According to our study, the spatial distribution of the microbial composition and structure and soil characteristics of the sites in PNLC was associated with sunlight exposure gradients, elevation and plant cover, specifically, on the basis of the comparison of two pairs of sites (sclerophyllous forest–xerophytic shrubland and hygrophilous forest–dry sclerophyllous forest).

Hence, the significant influence of climatic variables, such as precipitation and soil physicochemical conditions associated with the C and N composition of the organic matter, was observed in the sclerophyllous forest in comparison with the xerophytic shrubland. This is related to higher humidity conditions and a slightly lower temperature in the south-facing sclerophyllous forest, in contrast with the xerophytic shrubland, which presents a higher sunlight exposure, less plant cover and less humidity [37]. These environmental variables were significant in accounting for the prokaryotic and fungal community structure (Figure 6a,b). Variables relating to organic matter composition, temperature seasonality and temperature annual range, typical environmental conditions in this type of forest, in which growth is restricted to the bottom of ravines, i.e., with more humidity and less light availability, were especially significant for hygrophilous forest soil microorganisms [38]. Consequently, a higher richness, diversity and evenness of ravine fungal species, consistent with the data on the Central Chile template and coastal forests [69,70], was observed.

Slope, temperature seasonality and C/N were some of the significant variables for prokaryotes in the dry sclerophyllous forest. C/N, temperature annual range and temperature seasonality were significant for fungi. Although Chilean palm trees (*Jubaea chilensis*) in the dry sclerophyllous forest and hygrophilous forest are not predictors of the type of plant communities, since they are found in all habitats of Valle de Ocoa [71], the results showed a large Chytridiomycota and Mucoromycota fungal relative abundance that might be related to the presence of palm trees, which is a plant species found in both sites.

### 4.2. Variation in the Microbial Community and Its Association with the Environmental Conditions of the Plant Communities

There is a relationship between the microbial community structure in PNLC and plant distribution, where a greater differentiation between the bacterial and fungal communities in soils associated with the xerophytic shrubland and the hygrophillous forest, respectively, is highlighted. The comparison at higher taxonomical levels indicates that, in particular, soil fungi show more specificity associated with the plant communities that we have studied in PNLC.

In general, the bacterial phyla Proteobacteria, Actinobacteria and Acidobacteria comprised the most representative bacterial composition of soils, which is consistent with former studies of the Mediterranean semi-arid soils of PNLC [24]. The bacterial phyla Gemmatimonadetes, Bacteroidetes and Verrucomicrobia are frequently found in PNLC soil. The phylum Gemmatimonadetes is a cosmopolitan bacterial phylum, the distribution of which is related to the low water content of xeric soils [72]. Additionally, bacteria related with the bacterial phyla Bacteroidetes and Verrucomicrobia are commonly found in the rhizosphere, are involved in organic matter decomposition [73,74], and correlate with the soil physicochemical characteristics, such as pH and C/N ratio [75].

Certain individual microbial genera, in particular, showed a differential distribution associated with the plant communities; for instance, phylotype DA101 (Verrucomicrobia) [76] was the most abundant in the sclerophyllous forest and xerophytic shrubland, followed by the genera Rhodoplanes (Proteobacteria) in the sclerophyllous forest and dry sclerophyllous forest when comparing it with the other two sites (hygrophilous forest and xerophytic shrubland). Phylotype DA101 corresponds to Ca. Udaeobacter copiosus [76], a globally ubiquitous bacterium in a wide range of soils [75]. Rhodoplanes is a potentially phototrophic [77] and nitrogen-fixing bacteria [78]; Catania et al. (2022) reported that Rhodoplanes is a rare soil bacterium that has been detected in the soils of Spain, Portugal and Italy, associated with extremely dry and low-nutrient conditions [79], which are ecofunctional characteristics that allow them proliferate in Mediterranean semi-arid soils [80,81].

The phylum Ascomycota was the most abundant fungi in all of the sites, where its contribution to the total percentage of fungi in the sclerophyllous forest and dry sclerophyllous forest in PNLC stands out. In general, Ascomycota fungi play an important role in organic matter decomposition, the formation of humus, soil nutrient recycling and, additionally, are mutualists and parasites [82]. Furthermore, the significant relative abundance of Chytridiomycota and Mucoromycota in the hygrophilous forest and dry sclerophyllous forest suggests that these might be site-specific phyla of those plant communities, at a higher altitude, potentially favoring plant development in this type of environment in PNLC. The Chytridiomycotas group of fungi inhabits semi-arid soils [83] and show extreme cold and heat tolerance [84], while the saprophytic fungi of the phylum Mucoromycota are present in the soil and compost [85]. These correspond to endophytic fungal groups at the base of the phylogenetic tree of fungi [86] that might be adapted to specific environmental conditions that favor palm tree growth in the hygrophilous forest and the dry sclerophyllous forest.

Our research is consistent with the results reported by Egidi et al. (2019), which found high-specificity fungal OTUs when comparing four grassland (mild, mountain, coastal and arid) bioregions in Australia, the community structure of which was mainly associated with edaphic variables, such as soil organic carbon, pH, phosphorous, gravel, magnesium, manganese, copper and iron, as well as the distance between sites [87].

The distribution of the edaphic fungal and bacterial communities in PNLC is related with Mediterranean environmental conditions and potential ecological interactions with the endemic vegetation of the study area. Moreover, there is a higher fungal degree of specificity and potential endemism in PNLC compared with prokaryotes, since there are more than 37% unassigned groups and 1.8% unclassified groups in the databases in the case of fungi, in comparison with 3% of unassigned prokaryotic sequences. However, a high percentage of unassigned sequences has been reported for eukaryotes using ITS amplicons [88] and for functional fungal guilds, reaching up to 38.6% [89].

### 4.3. Key Microbial Taxa, Potential Functions and Their Relation with the Different Plant Communities of PNLC

In our study, the microbial function prediction indicates the presence of functional groups of the nitrogen cycle, among other biogeochemical cycles, which has been linked to forests with high plant species richness [90]—for example, nitrate reduction communities, such as Rhodoplanes [91] and ammonia-oxidizing archaea in the sclerophyllous forest, xerophytic shrubland and dry sclerophyllous forest. In general, the Archaea domain was not abundant in the PNLC soil; however, the ammonia-oxidizing archaea were mostly found in the sclerophyllous forest soil. This might be related to N reduction in both plant communities, since it has been suggested that the soil C/N ratio was the best individual predictor of archaea relative abundance and that dominance decreases in high inorganic N availability conditions, perhaps due to competitive interactions with nitrifying bacteria [92]. The foregoing is consistent with studies where an increased presence of archaea in north-facing slopes (shade) in the southern Alps in northern Italy has been observed [2].

Furthermore, our study found a predominance of microbial groups of the soil rare biosphere, such as phylum TM7 (Saccharibacteria) with Chlorobi [93], which are specific to the hygrophilous forest microbial community in comparison with the dry sclerophyllous forest microbial community. In the xerophytic shrubland, Chloroflexi [94] and Cyanobacteria [95], photosynthetic organisms that might be related to this site due to their higher sunlight exposure, were the most abundant prokaryotic phyla. The phylum Actinobacteria, along with the genera Pseudonocardia and Mycobacterium, which are closely related taxa that have been associated with a higher nutrient absorption [96], plant growth [97] and nitrogen fixation [98], share predominance in the hygrophilous forest and dry sclerophyllous forest soils.

Regarding fungi, saprophytic fungi, decomposers associated with the carbon cycle, were predominantly found, which is consistent with the results reported by research conducted in Norway’s beech tree forests [99,100] and in Mediterranean zones [101].

In the PNLC soil, the phylum Ascomycota, with the genera Alternaria, Rutstroemia, Cladosporium, Nothophoma and Zopfiella, among others, was mainly found in the grasslands and shrublands of arid environments which are associated with processes related with soil stability and plant biomass decomposition in this type of ecosystem [101]. Also, in the case of decomposer fungi, it was observed that the increase in the C/N ratio was related to a higher fungal diversity than bacterial diversity [102], which accounts for the larger number of specialist fungi in the hygrophilous forest, followed by the dry sclerophyllous forest and sclerophyllous forest. The genera Cladophialophora, described in different geographical regions; Hormonema, an opportunistic plant pathogen, mainly found in the phyllosphere [103,104]; and Coniochaeta, described as a tree pathogenic fungus [105], were observed in the dry sclerophyllous forest [106]. In particular, a higher relative abundance of the fungal community of the genera Solicoccozyma [107] of the phylum Basidiomycota was found in the dry sclerophyllous forest, hygrophilous forest and in a sample spot in the sclerophyllous forest.

The study of the soil fungal community indicates potential plant–fungal associations, such as in the case of the genus Humicola, which has been described as ascomycetes associated with ectomycorrhizas in the Tasmanian wet sclerophyllous forests [108], or the genera Dothideomycetes and Sordariomycetes, commonly found in tropical and subtropical palm trees [109].

In addition, the genus Mortierella spp. saprophytic and decomposer fungi, highly valuable for agricultural soils since they promote plant growth by way of phosphorous and iron bioavailability, were found in PNLC. These are also present in hostile environments [110] such as nutrient-poor (oligotrophic) soils, bare rocks, sandstone, dunes and areas destroyed by wildfires [111,112].

## 5. Conclusions

The microbial community structure, including prokaryotes and fungi, is spatially distributed in relation with a toposequence associated with Parque Nacional La Campana (PNLC)’s orientation and vegetation. In particular, the influence of the topoclimate conditions on the microbial community structure was more evident in contrasting sites, such as the xerophytic shrubland and hygrophilous forest. Both fungi and prokaryotes are dominated by genera of the rare biosphere in the hygrophilous forest and the dry sclerophyllous forest, which supports our hypothesis on the structure and role of microbial communities in PNLC. Specifically, each site presented dominant communities, highlighting the following: Crenarchaeote, Humicola and Mortierella were dominant in the sclerophyllous forest; Chloroflexi, Cyanobacteria and Alternaria in the xeric scrubland; Solicoccozyma in the dry sclerophyllous forest; and Cladophialophora in the hygrophilous forest. A great presence of Chytridiomycota and Mucoromycota fungi in the sclerophyllous dry forest and hygrophilous forest could be related to the presence of Chilean palms (*Jubaea chilensis*).

The potential functional prediction of microbial communities indicates the presence of functional groups associated with the nitrogen cycle, particularly those involved in nitrate reduction and ammonia oxidation, with a notable prevalence in the sclerophyllous forest and xerophytic scrub. The prevalence of saprophytic and decomposer fungi indicates a pivotal function in the decomposition of organic matter and the recycling of soil nutrients, a conclusion that is consistent with studies conducted in Mediterranean regions and temperate forests.

The greater diversity and specificity of fungi in hygrophytic forests compared to other vegetation types underscores the pivotal role of edaphic and climatic conditions in structuring fungal communities. The abundance of fungal genera such as Solicoccozyma, Humicola, Mortierella and others reflects potential plant–fungus associations and adaptations to specific environmental conditions in PNLC.

Further studies on the contribution of microorganisms are required to understand the role of diversity and microorganisms’ functions on the soil, so as to develop conservation strategies and the possible restoration of edaphic ecosystems affected by human activity.

## Figures and Tables

**Table 1:** Sampling points.

ID	Site	Slope Facing or Aspect	Geographic Location (Latitude)	Geographic Location (Longitude)	Elevation (masl)	Collection Date
SF1	Sclerophyllous forest	South	33°0′12.13″ S	71°7′24.62″ O	396	January 2020
SF2	Sclerophyllous forest	South	33°0′12.28″ S	71°7′25.17″ O	391	January 2020
SF3	Sclerophyllous forest	South	33°0′12.34″ S	71°7′26.40″ O	383	January 2020
SF4	Sclerophyllous forest	South	33°0′12.39″ S	71°7′25.82″ O	390	January 2020
XS1	Xerophytic shrubland	North	33°0′16.82″ S	71°7′28.00″ O	410	January 2020
XS2	Xerophytic shrubland	North	33°0′16.65″ S	71°7′27.32″ O	407	January 2020
XS3	Xerophytic shrubland	North	33°0′16.40″ S	71°7′27.80″ O	405	January 2020
XS4	Xerophytic shrubland	North	33°0′17.19″ S	71°7′27.43″ O	413	January 2020
HF1	Hygrophilous forest	V-shaped valley	32°57′13.86″ S	71°5′26.47″ O	590	February 2021
HF2	Hygrophilous forest	V-shaped valley	32°57′14.28″ S	71°5′26.84″ O	587	February 2021
HF3	Hygrophilous forest	V-shaped valley	32°57′14.63″ S	71°5′25.78″ O	585	February 2021
HF4	Hygrophilous forest	V-shaped valley	32°57′14.95″ S	71°5′26.46″ O	589	February 2021
DSF1	Dry sclerophyllous forest	West	32°57′14.14″ S	71°5′23.46″ O	621	February 2021
DSF2	Dry sclerophyllous forest	West	32°57′13.73″ S	71°5′23.92″ O	618	February 2021
DSF3	Dry sclerophyllous forest	West	32°57′14.51″ S	71°5′23.69″ O	624	February 2021
DSF4	Dry sclerophyllous forest	West	32°57′14.38″ S	71°5′23.02″ O	626	February 2021

## Data Availability

The original contributions presented in the study are included in the article/Supplementary Materials; further inquiries can be directed to the corresponding author/s.

## Supplementary Materials

The following supporting information can be downloaded at: https://www.mdpi.com/article/10.3390/microorganisms12081569/s1, Figure S1: Rarefaction curves for fungi sequences and Figure S2: Rarefaction curves for fungi prokaryotes, Table S1: Environmental and soil physico chemical conditions, Table S2: Per-sample sequence counts, Table S3: Significance regarding each environmental variables from the RDA analysis, Table S4: DeSeq analysis: identification of differentially represented ASVs in the microbial communities in the soil of plant communities in the xerophytic shrubland versus sclerophyllous forest and Table S5: DeSeq analysis: identification of differentially represented ASVs in the microbial communities in the soil of plant communities in the hygrophilous forest versus dry sclerophyllous forest.
