# Supplementary figures and images for: Microbial Diversity of Soil in a Mediterranean Biodiversity Hotspot: Parque Nacional La Campana, Chile
Source: Microorganisms. 2024 Jul 31;12(8):1569. doi: 10.3390/microorganisms12081569 (PMC11356564; doi:10.3390/microorganisms12081569)

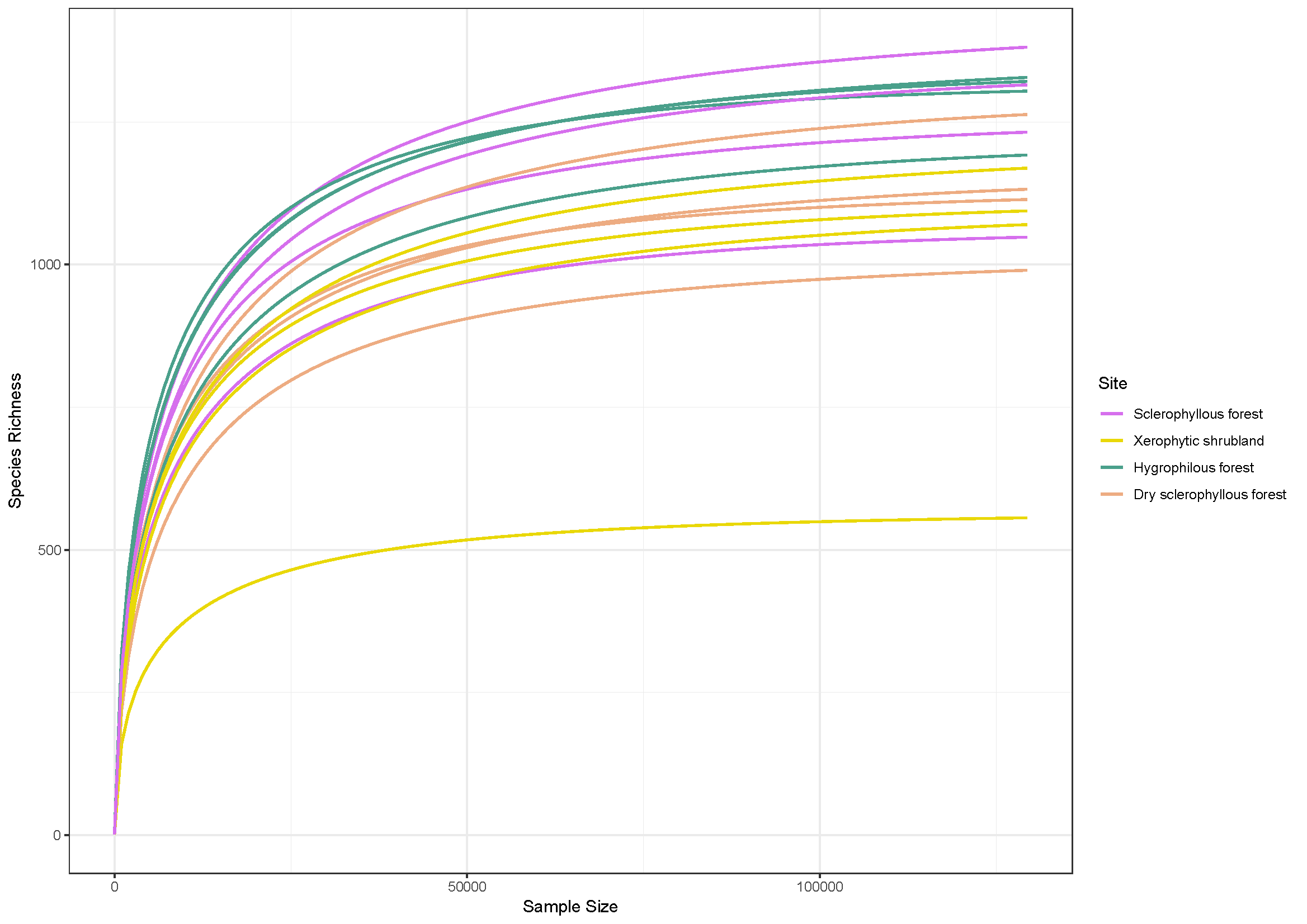

Supplement: Supplementary file 1 [file microorganisms-12-01569-s001.zip › S1.tif]

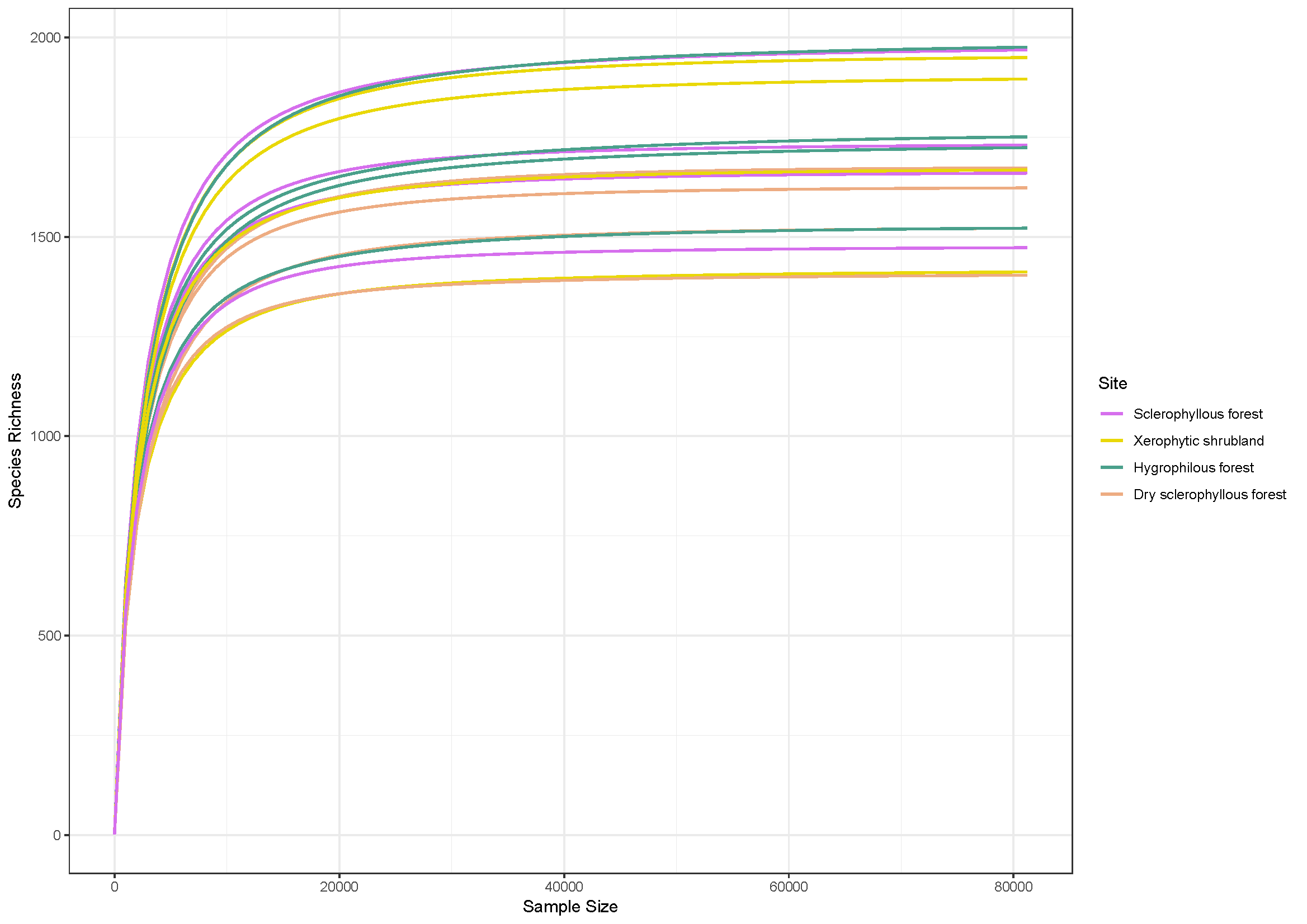

Supplement: Supplementary file 1 [file microorganisms-12-01569-s001.zip › S2.tif]
